# Bioinformatic-based genetic characterizations of neural regulation in skin cutaneous melanoma

**DOI:** 10.3389/fonc.2023.1166373

**Published:** 2023-06-19

**Authors:** Fengdi Wang, Fanjun Cheng, Fang Zheng

**Affiliations:** ^1^ Institute of Hematology, Union Hospital, Tongji Medical College, Huazhong University of Science and Technology, Wuhan, Hubei, China; ^2^ Department of Pediatrics, Union Hospital, Tongji Medical College, Huazhong University of Science and Technology, Wuhan, Hubei, China

**Keywords:** skin cutaneous melanoma, cancer–nerve crosstalk, neural regulation, bioinformatics, cancer immunotherapies

## Abstract

**Background:**

Recent discoveries uncovered the complex cancer–nerve interactions in several cancer types including skin cutaneous melanoma (SKCM). However, the genetic characterization of neural regulation in SKCM is unclear.

**Methods:**

Transcriptomic expression data were collected from the TCGA and GTEx portal, and the differences in cancer–nerve crosstalk-associated gene expressions between normal skin and SKCM tissues were analyzed. The cBioPortal dataset was utilized to implement the gene mutation analysis. PPI analysis was performed using the STRING database. Functional enrichment analysis was analyzed by the R package clusterProfiler. K-M plotter, univariate, multivariate, and LASSO regression were used for prognostic analysis and verification. The GEPIA dataset was performed to analyze the association of gene expression with SKCM clinical stage. ssGSEA and GSCA datasets were used for immune cell infiltration analysis. GSEA was used to elucidate the significant function and pathway differences.

**Results:**

A total of 66 cancer–nerve crosstalk-associated genes were identified, 60 of which were up- or downregulated in SKCM and KEGG analysis suggested that they are mainly enriched in the calcium signaling pathway, Ras signaling pathway, PI3K-Akt signaling pathway, and so on. A gene prognostic model including eight genes (GRIN3A, CCR2, CHRNA4, CSF1, NTN1, ADRB1, CHRNB4, and CHRNG) was built and verified by independent cohorts GSE59455 and GSE19234. A nomogram was constructed containing clinical characteristics and the above eight genes, and the AUCs of the 1-, 3-, and 5-year ROC were 0.850, 0.811, and 0.792, respectively. Expression of CCR2, GRIN3A, and CSF1 was associated with SKCM clinical stages. There existed broad and strong correlations of the prognostic gene set with immune infiltration and immune checkpoint genes. CHRNA4 and CHRNG were independent poor prognostic genes, and multiple metabolic pathways were enriched in high CHRNA4 expression cells.

**Conclusion:**

Comprehensive bioinformatics analysis of cancer–nerve crosstalk-associated genes in SKCM was performed, and an effective prognostic model was constructed based on clinical characteristics and eight genes (GRIN3A, CCR2, CHRNA4, CSF1, NTN1, ADRB1, CHRNB4, and CHRNG), which were widely related to clinical stages and immunological features. Our work may be helpful for further investigation in the molecular mechanisms correlated with neural regulation in SKCM, and in searching new therapeutic targets.

## 1 Introduction

Skin cutaneous melanoma (SKCM) represents one of the deadliest types of skin cancer in the world, which causes over 75% of skin cancer deaths. More recent discoveries have greatly improved the prognosis for patients with metastatic melanoma. The most promising therapies include the BRAF inhibitors and immune checkpoint blockers (ICBs). The BRAF inhibitors show effects on approximately half of melanoma patients with BRAF mutation, but a majority of patients develop secondary resistance within a relatively short time (1). Several ICBs have been approved for melanoma, including the cytotoxic T lymphocyte-associated protein 4 (CTLA-4) antibody ipilimumab and two programmed cell death protein 1 (PD-1) antibodies, nivolumab and pembrolizumab (2). Although these ICBs greatly improved the survival rate of stage IV melanoma patients, a significant proportion of patients remain no response to them and a part of responding patients show secondary resistance (3, 4). Therefore, the mechanisms of melanoma progression and new targets need to be further investigated.

Over the past 10 years, an increasing number of studies have revealed that nerve influenced the incidence and progression of cancers by affecting DNA mutations and oncogene-related signaling, modulating tumor-related immune responses, and promoting tumor growth and metastasis. In turn, the cancer tissues could induce the neoneurogenesis and axonogenesis, which suggests that the crosstalk between cancer and nerve plays an important role in the progression of cancer (5, 6). Such evidence has been identified in multiple cancer types including prostate, gastric, pancreatic, breast, colon, ovary, and head and neck cancers (7–15). In melanoma, sensory nerves have been found within the melanoma microenvironment and affected melanoma progression (16). The sympathetic nervous system also regulates melanoma biology, proven by a series of research indicating that α- and β-adrenergic receptors were expressed in human melanoma tissues and cell lines (17, 18). Moreover, nerve as a vital participator in the tumor microenvironment (TME) exerts influence over tumor biology not only by directly interacting with cancer cells but also indirectly with immune cells and stromal cells that subsequently impinge on tumor biology. In particular, neuroendocrine and neuronal pathways are involved in the control of immune responses in TME (19). These bring the researchers concentrating on the complex cancer–nerve crosstalk to the forefront, hopefully to overcome the difficulties in cancer immunotherapies. However, systematic analysis of genes associated with cancer–nerve interactions in SKCM on melanoma biology and patient outcomes has not been fully has not been fully conducted.

We herein identified 66 cancer–nerve crosstalk-associated genes and analyzed their gene expression and mutation signatures in tumor samples from SKCM patients in The Cancer Genome Atlas (TCGA). Then, we analyzed the prognostic values of cancer–nerve crosstalk-associated genes and correlations of these genes with clinical and immunological features. Our bioinformatics analysis may enlighten new inspiration in SKCM-targeted immunotherapies.

## 2 Methods

### 2.1 Identification of cancer–nerve crosstalk-associated genes

A total of 66 cancer–nerve crosstalk-associated genes were identified from previous references (20–22), and these genes are displayed in **Supplementary Table 1**.

### 2.2 Data acquisition and preprocessing

Gene expression profiles of 470 SKCM patients from the TCGA portal and the normal skin gene expression data of 812 samples extracted from the GTEx portal were obtained from UCSC XENA (https://xenabrowser.net/datapages/), which has recomputed all expression raw data based on a standard pipeline to minimize differences from distinct sources, thus eliminating the batch effects. Level 3 HTSeq-Fragments Per Kilobase Million (FPKM) data were then transformed into TPM (transcripts per million reads) for unpaired comparison and further analysis. Wilcoxon rank sum test was used to analyze the expression of cancer–nerve crosstalk-associated genes in non-paired samples. The clinical information of 472 melanoma patients was downloaded from the TCGA data portal (https://tcga-data.nci.nih.gov/tcga/). In addition, the GSE19234 (*n* = 44) (https://www.ncbi.nlm.nih.gov/geo/query/acc.cgi?acc=GSE19234) and the GSE59455 (*n* = 141) (https://www.ncbi.nlm.nih.gov/geo/query/acc.cgi?acc=GSE59455) were derived from the Gene Expression Omnibus (GEO) database for validation of prognostic genes. Use the R package ggplot2 for data processing, analysis, and visualization.

### 2.3 Mutation analysis of cancer–nerve crosstalk-associated genes

The cBioPortal for Cancer Genomics (http://cbioportal.org) integrated information of somatic mutations, DNA copy-number alterations (CNAs), mRNA and microRNA (miRNA) expression, DNA methylation, protein abundance, and phosphoprotein abundance from multiple public datasets including the Cancer Cell Line Encyclopedia (CCLE) and TCGA (23). We utilized the cBioPortal to implement the cancer–nerve crosstalk-associated gene mutation analysis and visualization.

### 2.4 Functional enrichment and protein–protein interaction analysis

Gene Ontology (GO) analysis and Kyoto Encyclopedia of Genes and Genomes (KEGG) analysis of the cancer–nerve crosstalk-associated genes were analyzed and visualized by the R package clusterProfiler and GOplot. The STRING database (https://string-db.org/) is an online database for searching known and predicted unknown protein–protein interactions (PPIs), which can be applied to more than 5,000 species, 24 million proteins, and more than 20 million PPI links (24). PPI analysis of cancer–nerve crosstalk-associated genes was carried out using the STRING database.

### 2.5 Construction and validation of the prognostic model

The expression of cancer–nerve crosstalk-associated genes in the TCGA cohort was used as an independent variable to perform univariate Cox regression analysis. Then, genes with *p* < 0.05 entered the least absolute shrinkage and selection operator (LASSO) regression by 10-fold cross-validation to further eliminate redundant genes using the R package glmnet. Genes with non-zero coefficient were deemed as prognostic genes. Next, a risk score map, a status map of overall survival (OS), and a heatmap of prognostic genes were created. The risk score of each patient in the TCGA and two validation cohorts according to the LASSO regression coefficient was calculated. Then, given that the prognosis of SKCM co-depends on multiple clinical and molecular features, the prognostic genes were integrated with other clinical factors (T, N, M stage, age, ulceration, Clark level, and Breslow depth) to construct a nomogram. Univariate and multivariate Cox regression analyses were used for the nomogram model to determine whether the prognostic genes were independent from other clinical parameters. The R package rms was used to build a calibration curve for the OS nomogram model. The risk score of each patient was calculated. The median value of the risk score was used as a cutoff to divide the patients into high- and low-risk groups. Log-rank regression was used to test Kaplan–Meier survival analysis and to compare the OS differences between the high- and low-risk-score group of each dataset. Time-ROC analysis was performed to evaluate the accuracy of the prediction by the R package timeROC.

### 2.6 Gene expression profiling interactive analysis

Gene expression profiling interactive analysis (GEPIA) is an online dataset (http://gepia2.cancer-pku.cn/#index) that provides customizable functions such as tumor/normal differential expression analysis, profiling according to cancer types or pathological stages, patient survival analysis, similar gene detection, correlation analysis, and dimensionality reduction analysis (25). We used GEPIA to analyze and visualize the association of prognostic genes with SKCM clinical stage.

### 2.7 Immune infiltration analysis

Immune infiltration analysis of SKCM samples was performed by the ssGSEA method using the R package GSVA for 24 types of immune cells, including neutrophils, mast cells, eosinophils, macrophages, natural killer (NK) cells, CD56^dim^ NK cells, CD56bright NK cells, dendritic cells (DCs), immature DCs (iDCs), activated DCs (aDCs), plasmacytoid DCs (pDCs), T cells, CD8^+^ T cells, T helper cells (Th), Th1 cells, Th2 cells, Th17 cells, T follicular helper cells (Tfh), regulatory T cells (Treg), central memory T cells (Tcm), effector memory T cells (Tem), gamma delta T cells (Tgd), cytotoxic cells, and B cells. Based on the reported signature genes for the 24 types of immunocytes, the relative enrichment score of each immunocyte was quantified from the gene expression profile for each tumor sample. Spearman’s correlation coefficient analysis was performed to identify relationships of the prognostic genes with each type of lymphocyte. Gene Set Cancer Analysis (GSCA) (http://bioinfo.life.hust.edu.cn/GSCA/#/) is a database, uniquely providing gene set search and compiling scores by gene set enrichment analysis (GSEA) and gene set variation analysis (GSVA) to investigate correlations between immune infiltration and the integrated level of the expression of gene set (GSVA score) (26). Immunogenomic analysis of GSCA was performed by the R package ImmuCellAI with 24 immune cells including DCs, B cells, monocytes, macrophages, NK cells, neutrophils, CD4^+^ T cells, CD8^+^ T cells, NK T cells, Tgd, CD8^+^ naïve cells, CD8^+^ naïve cells, cytotoxic cells, exhausted T cells (Tex), type 1 regulatory T cells (Tr1), natural Treg cells (nTreg), induced Treg cells (iTreg), Th1, Th2, Th17, Tfh, Tcm, Tem, and mucosal associated invariant T cells (MAIT). We used GSCA to compare the correlations between immune infiltration with the GSVA score of the prognostic gene set by Spearman’s correlation coefficient analysis.

### 2.8 Correlation of prognostic genes with immune checkpoint genes

Spearman’s correlation coefficient analysis was used to determine the association of prognostic genes with immune checkpoint genes, including negative immune regulator PDCD1 (encoding PD-1), CD274 [encoding programmed cell death-ligand 1 (PD-L1)], CTLA4 (encoding CTLA 4), LAG3 (encoding lymphocyte activation gene-3), TIGIT (encoding T-cell immunoglobulin and ITIM domain), HAVCR2 (encoding T-cell immunoglobulin and mucin-domain containing-3), VSIR (encoding V-domain Ig suppressor of T cell activation), and positive regulator TNFRSF4 (encoding OX40) (27).

### 2.9 Gene set enrichment analysis

GSEA is an analytical method that determines differences between two phenotypes based on a previously defined gene set (https://www.gsea-msigdb.org/gsea/index.jsp) (28). GSEA was used in order to elucidate the significant function and pathway differences between the high and low expression of prognostic genes. Gene set permutations were performed 5,000 times for each analysis. The study chose c2.cp.v7.2.symbols.gmt [Curated] in the MSigDB Collections as the reference gene collection. An adjusted *p* < 0.05, FDR < 0.25, and normalized enrichment score (|NES|) > 1 was considered as significant enrichment.

## 3 Results

### 3.1 Study protocol

The schematic diagram of the study protocol is shown in **Supplementary Figure 1**.

### 3.2 Expression and mutation profiles of cancer–nerve crosstalk-associated genes in SKCM

**Figure 1:**
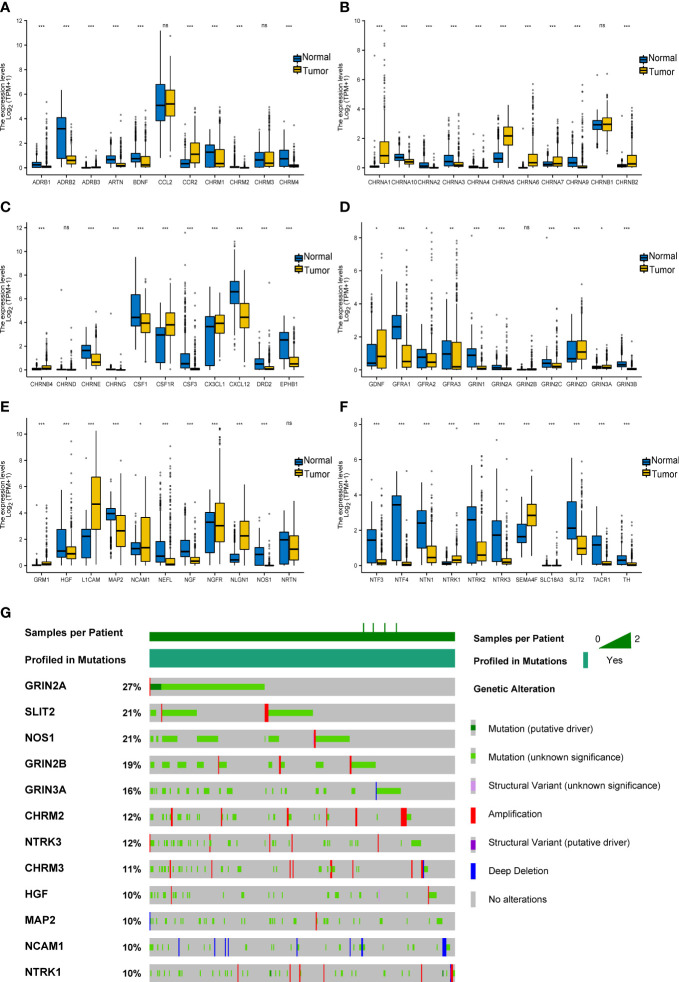
Expression and mutation of cancer–nerve crosstalk-associated genes in SKCM. **(A–F)** The expression of 66 cancer–nerve crosstalk-associated genes in SKCM and normal skin tissue. Wilcoxon rank sum test was used to analyze the expression of cancer–nerve crosstalk-associated genes in non-paired samples. **(G)** The landscape of the top 12 mutation rate of cancer–nerve crosstalk-associated genes in SKCM. ns, *p* > 0.05; *p < 0.05; **p < 0.01; ***p < 0.001. SKCM, skin cutaneous melanoma.

First, we explored the expression level of 66 cancer–nerve crosstalk-associated genes in SKCM tissues from the TCGA database, and the clustered heatmap is shown in **Supplementary Figure 2**. Then, we compared the expression of 66 cancer–nerve crosstalk-associated genes between SKCM tissues in the TCGA database and normal skin tissues in the GTEx (**Supplementary Table 2**). Forty of these cancer–nerve crosstalk-associated genes were significantly downregulated (*p* < 0.05), and 20 of these were significantly upregulated (*p* < 0.05) (**Figures 1A–F**).

Then, through the cBioPortal database, we summarized the incidence of genetic alteration of 66 cancer–nerve crosstalk-associated genes in SKCM patients including the DNA copy number alterations (CNAs) and somatic mutations. Cancer–nerve crosstalk-associated genes were altered in 375 of queried 444 samples (85%). The most common type of mutation was missense mutation. We showed the top 12 genes with the highest mutation rate: GRIN2A (27%), SLIT2 (21%), NOS1 (21%), GRIN2B (19%), GRIN2A (16%), CHRM2 (12%), NTRK3 (12%), CHRM3 (11%), HGF (10%), MAP2 (10%), NCAM1 (10%), and NTRK1 (10%) (**Figure 1G**). However, most of these alterations were with unknown significance. Genetic alterations with known significance concentrated on GRIN2A, EPHB1, and NTRK1, accounting for 5% of queried samples. Consistently, GRIN2A still had the highest mutation rate of 2.5%, followed by NTRK1 (2.3%) and EPHB1 (0.5%).

### 3.3 Functional enrichment and PPIs of cancer–nerve crosstalk-associated genes

**Figure 2:**
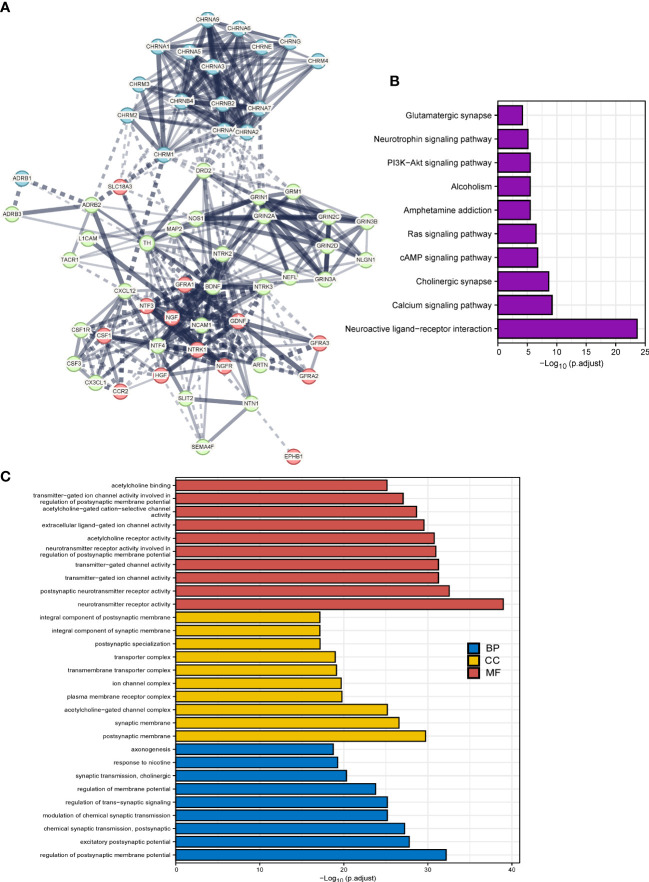
The functional enrichment analysis of cancer–nerve crosstalk-associated genes. **(A)** The PPIs of cancer–nerve crosstalk-associated genes. Three clusters were shown in different colors (red, light green, and blue) by k-means clustering. Line thickness indicated the strength of data support, and edges between clusters were shown in dotted lines. **(B)** The KEGG analysis of up- or downregulated cancer–nerve crosstalk-associated genes in SKCM. **(C)** The GO analysis of up- or downregulated cancer–nerve crosstalk-associated genes in SKCM. PPIs, protein–protein interactions; KEGG, Kyoto Encyclopedia of Genes and Genomes; BP, biological process; CC cellular component; MF, molecular function.

We implemented the PPI analysis of 66 cancer–nerve crosstalk-associated genes, and then k-means clustering gave three clusters shown in different colors (red, light green, and blue, **Figure 2A**). The results displayed an intricate interaction among all the genes and three clusters. Then, we focused on the functional enrichment of 60 up- or downregulated genes by KEGG pathway analysis and GO analysis. Through KEGG pathway analysis, we found that 60 up- or downregulated genes were mainly enriched in neuroactive ligand–receptor interaction, calcium signaling pathway, cholinergic synapse, cAMP signaling pathway, Ras signaling pathway, PI3K-Akt signaling pathway, and so on (**Figure 2B**), most of which are substantial oncologic signaling pathways. Meanwhile, through GO analysis, we found that 60 up- or downregulated genes were mainly enriched in a series of regulation of synaptic formation and function, axonogenesis, and so on (**Figure 2C**), which further confirmed that the functions of genes we queried indeed concentrated on neural regulations.

### 3.4 Construction and validation of the prognostic gene model

**Figure 3:**
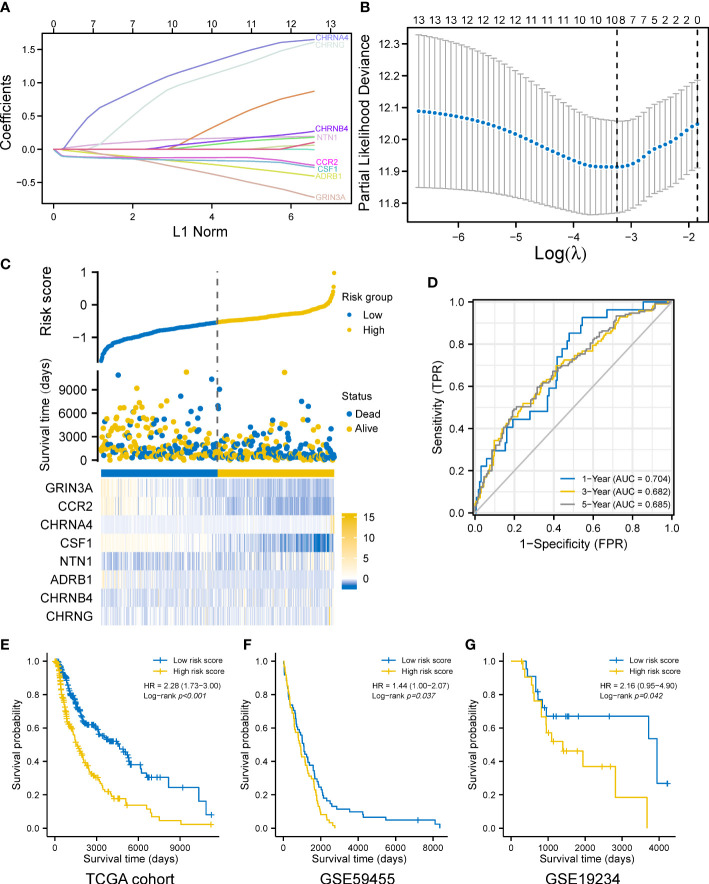
Construction of a prognostic gene model. **(A)** LASSO coefficient profiles of the eight prognostic cancer–nerve crosstalk-associated genes. **(B)** Plots of the 10-fold cross-validation error rates. **(C)** Distribution of risk score, survival status, and the expression of eight cancer–nerve crosstalk-associated genes in SKCM. **(D)** The ROC curve of measuring the predictive value. **(E)** Overall survival curves for SKCM patients in the high-/low-risk-score group. **(F)** Overall survival curve for 141 SKCM patients of GSE59455 in the high-/low-risk-score group. **(G)** Overall survival curve for 44 SKCM patients of GSE19234 in the high-/low-risk-score group. Log-rank test was used for comparing the overall survival between groups. ROC, receiver operating characteristic curve; FPR, false-positive rate; TPR, true-positive rate; AUC, area under curve.

We explored the prognostic values of nerve–cancer crosstalk-associated genes. Univariate Cox analysis of 66 nerve–cancer crosstalk-associated genes was carried out, and hazard ratio (HR) and *p*-value were calculated (**Supplementary Table 3**). Based on those genes with *p* < 0.05, which included GRIN3A, CCL2, CCR2, CHRNA4, CSF1, CSF1R, NTN1, NTF4, CXCL12, ADRB1, CHRNB4, CHRND, and CHRNG, we used LASSO regression analysis to further eliminate redundant genes (**Figures 3A, B**). Finally, a total of eight genes were included in the prognostic gene model, and risk score = GRIN3A * (−0.24220) + CCR2 * (−0.12696) + CHRNA3 * 1.09475 + CSF1 * (−0.15966) + NTN1 * 0.14374 + ADRB1 * (−0.16631) + CHRNB4 * 0.04553 + CHRNG * 0.89123. We calculated the risk score (**Supplementary Table 4**) for each SKCM patient with survival data in the TCGA cohort and divided them into the high- and low-risk-score group based on the median risk score. The risk score distribution, survival status, and the expression of eight genes are shown in **Figure 3C**. The AUCs of the 1-, 3-, and 5-year ROC curve were 0.704, 0.682, and 0.685, respectively (**Figure 3D**). The Log-rank test showed that the prognosis of the high-risk-score group was significantly worse than that of the low-risk-score group [with a median OS of 1,524 (1,315–2,030) days vs. 4,634 (3,139–6,164) days, *p* < 0.001] with an HR of 2.28 (95% CI 1.73–3.00) (**Figure 3E**). Furthermore, we verified the effectiveness of the prognostic gene model using external datasets, GSE59455 and GSE19234. The risk score of patients with survival data was calculated and the survival of high- and low-risk-score groups was compared by Kaplan–Meier plotter (data shown in **Supplementary Table 5**). The results showed that the model could predict the prognosis with the HR of 1.44 (95% CI 1.00–2.07, *p* = 0.037) for GSE59455 (**Figure 3F**), and the HR of 2.16 (95% CI 0.95–4.90, *p* = 0.042) for GSE19234 (**Figure 3G**).

### 3.5 Construction and validation of the prognostic nomogram

**Figure 4:**
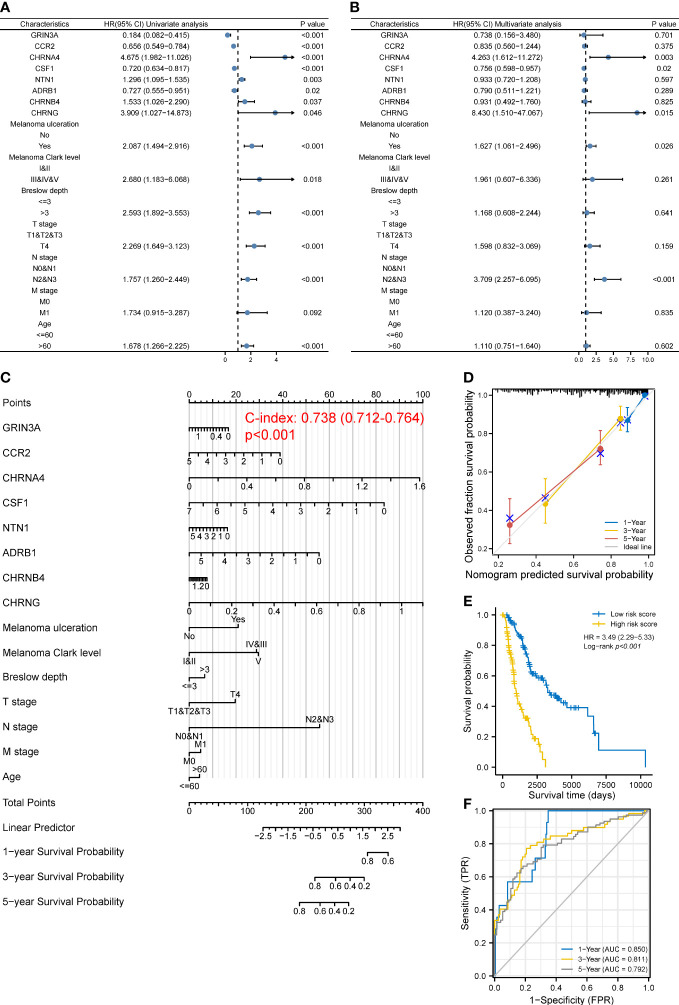
Construction of a predictive nomogram. **(A, B)** HR and *p*-value of the constituents involved in univariate **(A)** and multivariate **(B)** Cox regression considering clinical factors and eight prognostic cancer–nerve crosstalk-associated genes in SKCM. **(C)** Nomogram to predict the 1-, 3-, and 5-year overall survival rate of SKCM patients. **(D)** Calibration curve for the overall survival nomogram mode. The gray diagonal line represents the ideal nomogram. **(E)** Overall survival curves for SKCM patients in the high-/low-risk-score group. Log-rank test was used for comparing the overall survival between groups. **(F)** The ROC curve of measuring the predictive value of the nomogram.

Given that the prognosis of SKCM co-depends on multiple clinical and molecular features, we next incorporated both the common clinical characteristics of SKCM (T, N, M stage, age, ulceration, Clark level, and Breslow depth) and these eight prognostic genes in a model to establish a nomogram followed by univariate and multivariate analyses (**Figures 4A, B**), thus improving prognostic predictive power and looking for independent prognostic genes. The nomogram is shown in **Figure 4C**, with a C index of 0.738 (95% CI 0.712–0.764, *p* < 0.001). Results revealed that CHRNA4 (HR 4.263, 95% CI 1.612–11.272, *p* = 0.003), CHRNG (HR 8.430, 95% CI 1.510–47.067, *p* = 0.015), melanoma ulceration (HR 1.627, 95% CI 1.061–2.496, *p* = 0.026), and N2&3 stage (HR 3.709, 95% CI 2.257–6.095, *p* < 0.001) were independent poor prognostic factors. Moreover, the nomogram could predict the 1-, 3-, and 5-year survival rates close to the ideal model (**Figure 4D**). The risk score (**Supplementary Table 6**) for each TCGA-SKCM patient was calculated according to the results of multivariate analyses and divided them into the high-risk-score group and the low-risk-score group based on median risk score as cutoff value. Reasonably, the Log-rank test showed that the prognosis of the high-risk-score group was worse than that of the low-risk-score group [with a median OS of 996 (821–1,413) days vs. 3,266 (2,470–6,590) days, *p* < 0.001], and the HR (3.49, 95% CI 2.29–5.33) was higher than the prognostic gene model above (**Figure 4E**). The AUCs of the 1-, 3-, and 5-year ROC curve of this nomogram were 0.850, 0.811, and 0.792, respectively (**Figure 4F**), which were higher than the corresponding AUCs of the prognostic gene model based on LASSO regression above.

### 3.6 Relationships between prognostic genes and clinical stage of SKCM

**Figure 5:**
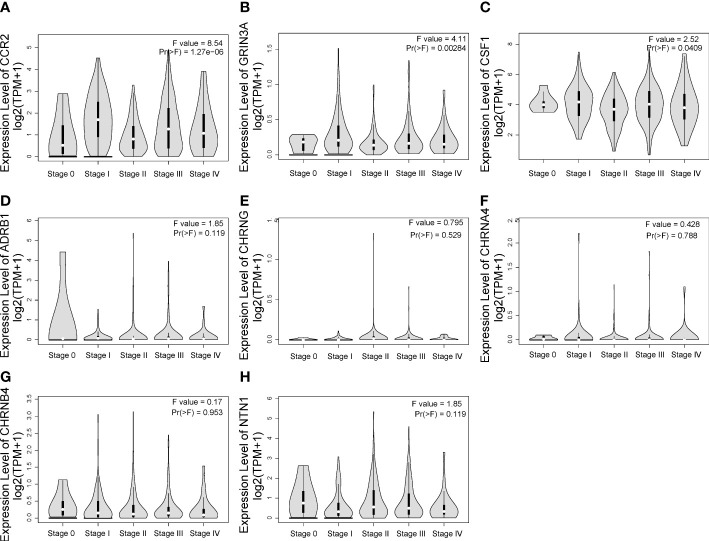
The relationship between eight prognostic cancer–nerve crosstalk-associated genes and SKCM clinical stage. Expression of CCR2 **(A)**, GRIN3A **(B)**, CSF1 **(C)**, ADRB1 **(D)**, CHRNG **(E)**, CHRNA4 **(F)**, CHRNB4 **(G)**, and NTN1 **(H)** in different stages in SKCM. One-way ANOVA was used for comparison of gene expression in different clinical stages.

One-way ANOVA was performed on GEPIA to evaluate the association between the expression of eight prognostic genes and clinical stage of SKCM. As shown in **Figures 5A–C**, CCR2 [*F* value = 8.54, Pr(>*F*) = 1.27e-06], GRIN3A [*F* value = 4.11, Pr(>*F*) = 0.00284], and CSF1 [*F* value = 2.52, Pr(>*F*) = 0.0409] were associated with clinical stage. However, we found no correlation between ADRB1 [Pr(>*F*) = 0.119], CHRNG [Pr(>*F*) = 0.529], CHRNA4 [Pr(>*F*) = 0.788], CHRNB4 [Pr(>*F*) = 0.953], and NTN1 [Pr(>*F*) = 0.119] with clinical stage of SKCM (**Figures 5D–H**).

### 3.7 Relationship between cancer–nerve crosstalk-associated genes and immune cell infiltration in SKCMs

Compared with other types of cancers, melanoma possessed the high mutational burdens, which increases both their immunogenicity and the infiltration of immune cells into the TME (29). Furthermore, in the process of tumor progression and metastasis, the reciprocal communication between immune and nerve correlates with poor prognosis (20, 30). Hence, we explored the association between the cancer–nerve crosstalk-associated genes and immune cell infiltration (**Supplementary Figure 3**), especially the eight prognostic genes.

**Figure 6:**
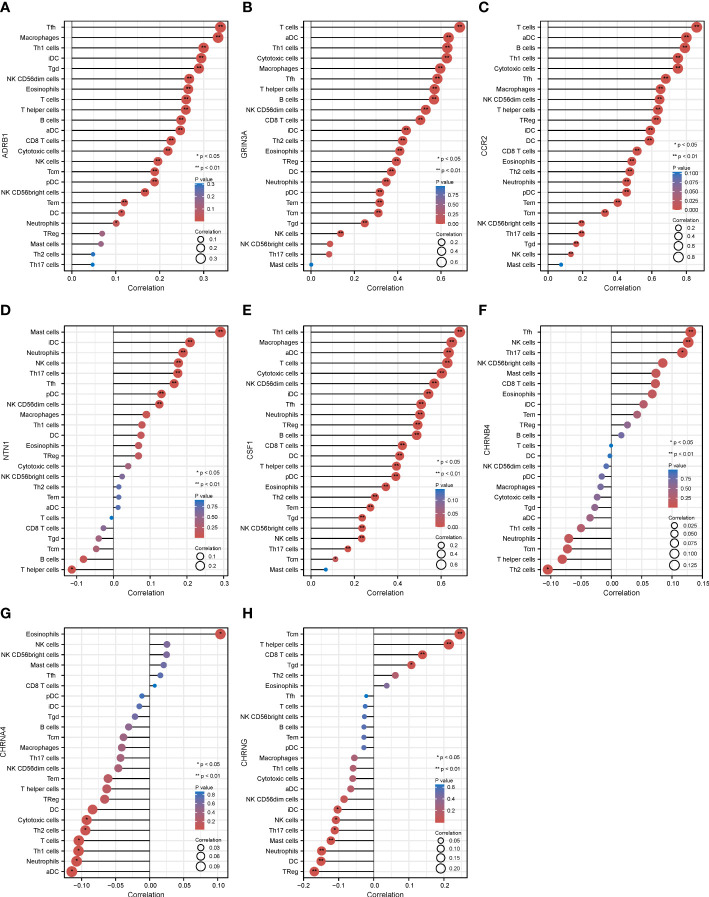
The relationship between eight prognostic cancer–nerve crosstalk-associated genes and immune cell infiltration in SKCM. Lollipop chart of the Spearman’s correlation between ADRB1 **(A)**, GRIN3A **(B)**, CCR2 **(C)**, NTN1 **(D)**, CSF1 **(E)**, CHRNB4 **(F)**, CHRN4A **(G)**, and CHRNG **(H)**, and 24 types of immune cells were shown. Spearman’s correlation coefficient analysis was used. iDC, immature DC; aDC, activated DC; pDC, plasmacytoid DC; Tfh, follicular helper T cells; Tgd, gamma delta T cells.

The eight prognostic genes were generally associated with various immune cells infiltrated in TME. Specifically, ADRB1 (**Figure 6A**) was significantly positively correlated with Tfh (*r* = 0.338, *p* = 0.01), macrophages (*r* = 0.333, *p* < 0.001), Th1 (*r* = 0.300, *p* < 0.001), and so on. GRIN3A (**Figure 6B**) was significantly positively correlated with T cells (*r* = 0.683, *p* < 0.001), aDC (*r* = 0.633, *p* < 0.001), Th1 cells (*r* = 0.629, *p* < 0.001), cytotoxic cells (*r* = 0.624, *p* < 0.001), macrophages (*r* = 0.597, *p* < 0.001), Tfh cells (*r* = 0.581, *p* < 0.001), T helper cells (*r* = 0.569, *p* < 0.001), B cells (*r* = 0.564, *p* < 0.001), and so on. CCR2 (**Figure 6C**) was significantly positively correlated with most immune cells expect for mast cells. NTN1 (**Figure 6D**) was significantly positively correlated with mast cells (*r* = 0.297, *p* < 0.001), iDC (*r* = 0.209, *p* < 0.001), and so on. CSF1 (**Figure 6E**) was also significantly positively correlated with most immune cells expect for mast cells. CHRNB4 and CHRNA4 (**Figures 6F, G**) was not strongly correlated with infiltrated immune cells (*r* < 0.2). CHRNG (**Figure 6H**) was significantly positively correlated with Tcm cells (*r* = 0.242, *p* < 0.001), T helper cells (*r* = 0.212, *p* < 0.001), and so on. In conclusion, our results showed that there is a close relationship between the prognostic cancer–nerve crosstalk-associated genes and immune cell infiltration (**Table 1**).

**Table 1:** The association between eight prognostic cancer–nerve–immune crosstalk-associated genes and immune cell infiltration.

	B cells	CD8^+^ T	Cytotoxic cells	DC	Macrophage	Mast cells	Neutrophil	NK	Th	Tcm	Tem	Treg
ADRB1	0.249^***^	0.226^***^	0.219^***^	0.113^*^	0.333^***^	0.066	0.101^*^	0.196^***^	0.259^***^	0.189^***^	0.120^*^	0.069
GRIN3A	0.564^***^	0.501^***^	0.624^***^	0.370^***^	0.597^***^	0.005	0.348^***^	0.140^**^	0.569^***^	0.311^***^	0.316^***^	0.390^***^
CCR2	0.790^***^	0.514^***^	0.750^***^	0.584^***^	0.644^***^	0.071	0.453^***^	0.130^**^	0.630^***^	0.327^***^	0.404^***^	0.624^***^
NTN1	−0.08	−0.03	0.04	0.07	0.09^*^	0.30^***^	0.19^***^	0.18^***^	−0.11^*^	−0.05	0.01	0.07
CSF1	0.63^***^	0.50^***^	0.68^***^	0.52^***^	0.77^***^	0.11^*^	0.57^***^	0.26^***^	0.52^***^	0.25^***^	0.37^***^	0.54^***^
CHRNB4	0.014	0.070	−0.024	−0.002	−0.014	0.078	−0.068	0.133^*^	−0.080	−0.072	0.045	0.023
CHRNA4	−0.031	0.008	−0.093^*^	−0.084	−0.039	0.024	−0.106^*^	0.024	−0.063	−0.039	−0.065	−0.064
CHRNG	−0.030	0.138^**^	−0.060	−0.150^**^	−0.053	−0.118^*^	−0.146^**^	−0.102^*^	0.212^***^	0.242^***^	−0.026	−0.0002

Spearman’s correlation was used to test the correlation coefficients. *p < 0.05; **p < 0.01; ***p < 0.001. DC, dendritic cells; NK, natural killer cells; Th, T helper cells; Tcm, central memory T cells; Tem, effector memory T cells; Treg, regulatory T cells.

**Figure 7:**
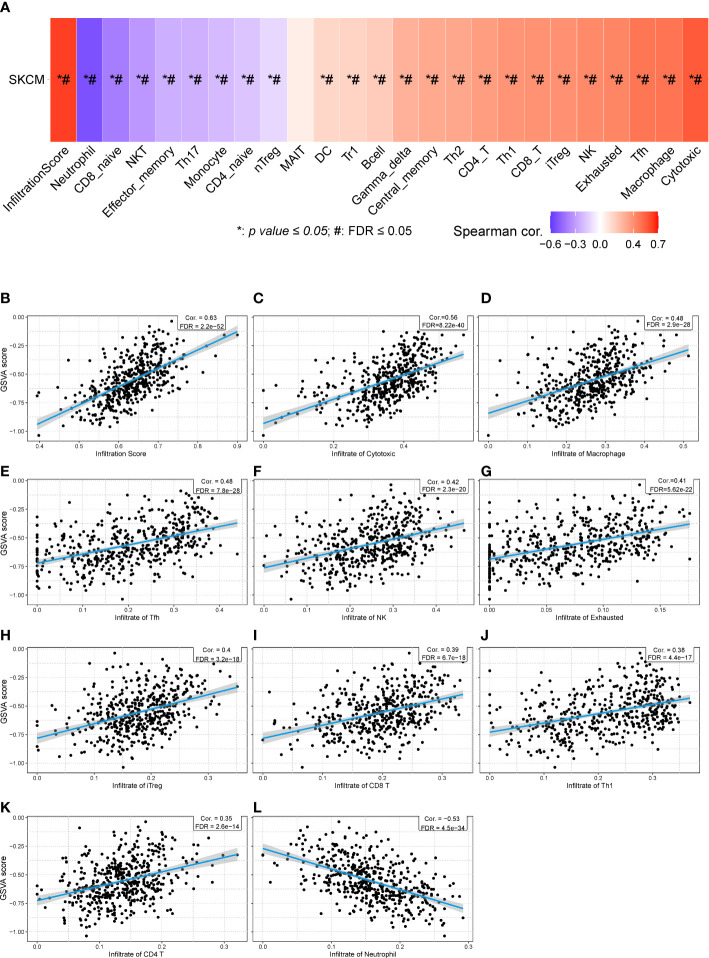
The relationships between GSVA score of eight prognostic cancer–nerve crosstalk-associated genes and immune cell infiltration in SKCM. **(A)** The summarization of the association between GSVA score of eight prognostic cancer–nerve crosstalk-associated genes and activities of 24 types of infiltrated immune cells in SKCM. The scatter plots of the relationships between the CSVA score of prognostic cancer–nerve crosstalk-associated genes and infiltrate score **(B)**, infiltrate of cytotoxic T cells **(C)**, macrophages **(D)**, Tfh cells **(E)**, NK cells **(F)**, exhausted T cells **(G)**, iTreg cells **(H)**, CD8^+^ T cells **(I)**, Th1 cells **(J)**, CD4^+^ T cells **(K)**, and neutrophils **(L)**, in SKCM. Spearman’s correlation coefficient analysis was used. The blue line was the linear fit and the gray area was the 95% confidence interval. iTreg, induced regulatory T cells.

Then, we calculated the GSVA score of the prognostic gene set including CHRNA4, CSF1, GRIN3A, CCR2, CHRNB4, ADRB1, NTN1, and CHRNG (**Figure 7A**), showing that the GAVA score was positively correlated with the infiltrate score [**Figure 7B**, *r* = 0.63, false discovery rate (FDR) < 0.001] and the infiltrate of cytotoxic cells (**Figure 7C**, *r* = 0.56, FDR < 0.001), macrophages (**Figure 7D**, *r* = 0.48, FDR < 0.001), Tfh cells (**Figure 7E**, *r* = 0.48, FDR < 0.001), NK cells (**Figure 7F**, *r* = 0.42, FDR < 0.001), exhausted T cells (**Figure 7G**, *r* = 0.41, FDR < 0.001), iTreg cells (**Figure 7H**, *r* = 0.40, FDR < 0.001), CD8^+^ T cells (**Figure 7I**, *r* = 0.39, FDR < 0.001), Th1 cells (**Figure 7J**, *r* = 0.380, FDR < 0.001), and CD4^+^ T cells (**Figure 7K**, *r* = 0.35, FDR < 0.001). The GAVA score was negatively correlated with neutrophils (**Figure 7L**, *r* = −0.53, FDR < 0.001). The results indicated that not only the expression of individual prognostic genes but also the integrated expression of gene set was associated with immune cell infiltration.

### 3.8 Relationship between cancer–nerve crosstalk-associated genes and immune checkpoint genes in SKCM

**Figure 8:**
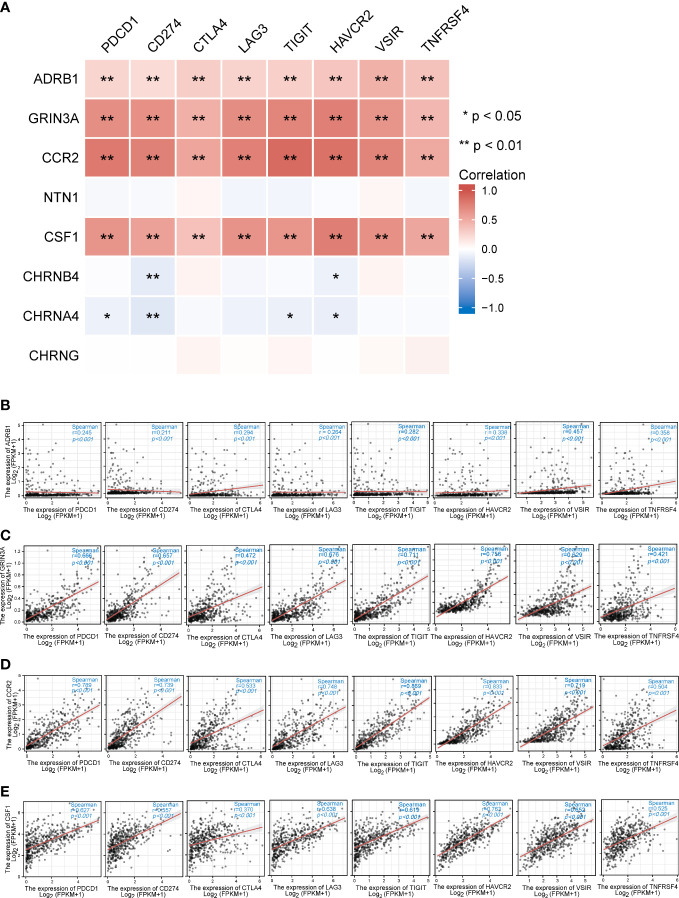
The relationships between eight prognostic cancer–nerve crosstalk-associated genes and immune checkpoint genes in SKCM. **(A)** The heatmap of the association between eight prognostic cancer–nerve crosstalk-associated genes and immune checkpoint genes in SKCM. The scatter plots of the relationships between the immune checkpoint genes and ADRB1 **(B)**, GRIN3A **(C)**, CCR2 **(D)**, and CSF1 **(E)** were shown. Spearman’s correlation coefficient analysis was used. The red line was the linear fit and the gray area was the 95% confidence interval.

Unprecedented advances have been made in melanoma treatment with the use of ICBs. However, responses are limited. We endeavored to research the relationship of prognostic genes with important immune checkpoint genes reported in SKCM, including PDCD1, CD274, CTLA4, LAG3, TIGIT, HANCR2, VSIR, and THFRSF4 (**Table 2**). It turned out that ADRB1, GRIN3A, CCR2, and CSF1 were universally associated with these immune checkpoint genes as shown in the heatmap (**Figure 8A**). **Figures 8B–E** presented the scatter plots and fitting lines of the relationship of each immune checkpoint gene with these four prognostic genes. The results indicated that these prognostic genes play a specific role in the expression pattern of immune checkpoint genes in SKCM and may be involved in the response of SKCM patients to ICBs.

**Table 2:** The association between eight prognostic cancer–nerve–immune crosstalk-associated genes and immune checkpoint genes.

	ADRB1	GRIN3A	CCR2	NTN1	CSF1	CHRNB4	CHRN4A	CHRNG
PDCD1	0.245^***^	0.663	0.789	−0.046	0.623^***^	−0.017	−0.092^*^	−0.005
CD274	0.212^***^	0.653	0.739	−0.039	0.551^***^	−0.129^**^	−0.163^***^	0.003
CTLA4	0.295^***^	0.471	0.532	0.055	0.364^***^	0.071	−0.041	0.059
LAG3	0.261^***^	0.672	0.747	−0.066	0.637^***^	−0.050	−0.089	0.014
TIGIT	0.282^***^	0.711	0.869	−0.069	0.619^***^	−0.036	−0.098^**^	0.056
HAVCR2	0.337^***^	0.754	0.829	−0.025	0.761^***^	−0.092^*^	−0.116^**^	0.006
VSIR	0.457^***^	0.629	0.719	0.054	0.652^***^	0.060	−0.032	0.041
TNFRSF4	0.357^***^	0.421	0.501	−0.056	0.521^***^	−0.028	−0.035	0.083

Spearman’s correlation coefficient analysis was performed to measure relationships of each prognostic gene with the immune checkpoint genes. *p < 0.05; **p < 0.01; ***p < 0.001.

### 3.9 Functions of independent prognostic genes CHRN4A and CHRNG

**Figure 9:**
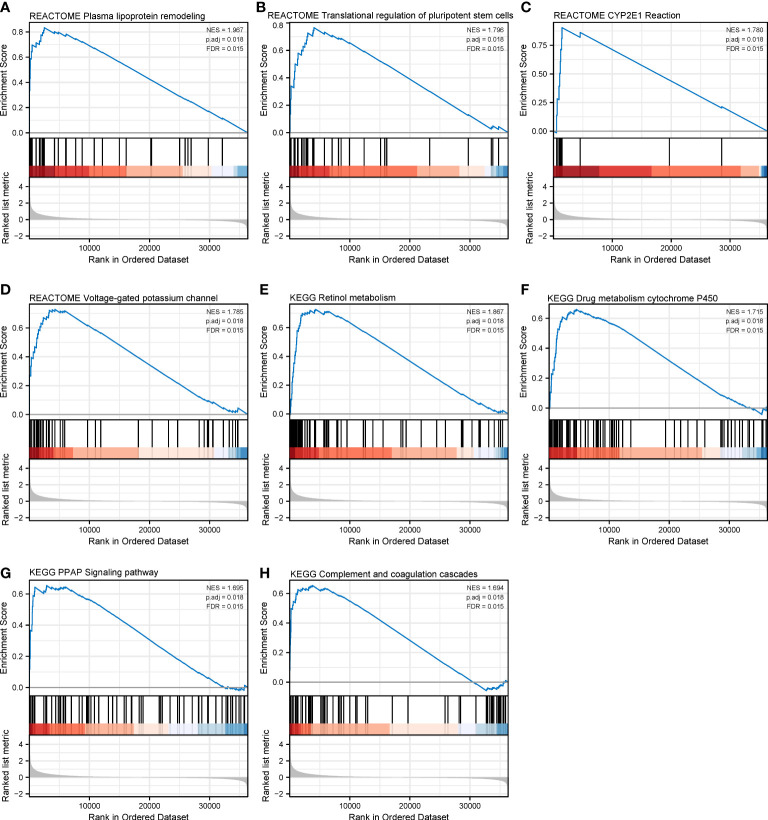
Gene set enrichment analysis of CHRNA4 in SKCM. The expression of CHRNA4 positively correlated with plasma lipoprotein remodeling **(A)**, transcriptional regulation of pluripotent stem cells **(B)**, CYP2E1 reactions **(C)**, and voltage-gated potassium channels **(D)** in Reactome gene sets. The expression of CHRNA4 positively correlated with retinol metabolism **(E)**, drug metabolism cytochrome P450 **(F)**, PPAR signaling pathway **(G)**, and complement and coagulation cascades **(H)** in KEGG gene sets.

We have identified CHRNA4 and CHRNG as independent poor prognostic factors based on the results of multivariate Cox regression. Then, we tried to analyze their functions by performing GSEA to identify the key pathways related to CHRNA4 and CHRNG. GSEA found that the significantly enriched pathways involved in high CHRNA4 expression included plasma lipoprotein remodeling (**Figure 9A**), transcriptional regulation of pluripotent stem cells (**Figure 9B**), CYP2E1 reactions (**Figure 9C**), and voltage-gated potassium channels (**Figure 9D**) in Reactome gene sets. Meanwhile, results also showed the following enriched KEGG pathways in connection with high CHRNA4 expression: retinol metabolism (**Figure 9E**), drug metabolism cytochrome P450 (**Figure 9F**), PPAR signaling pathway (**Figure 9G**), and complement and coagulation cascades (**Figure 9H**).

**Figure 10:**
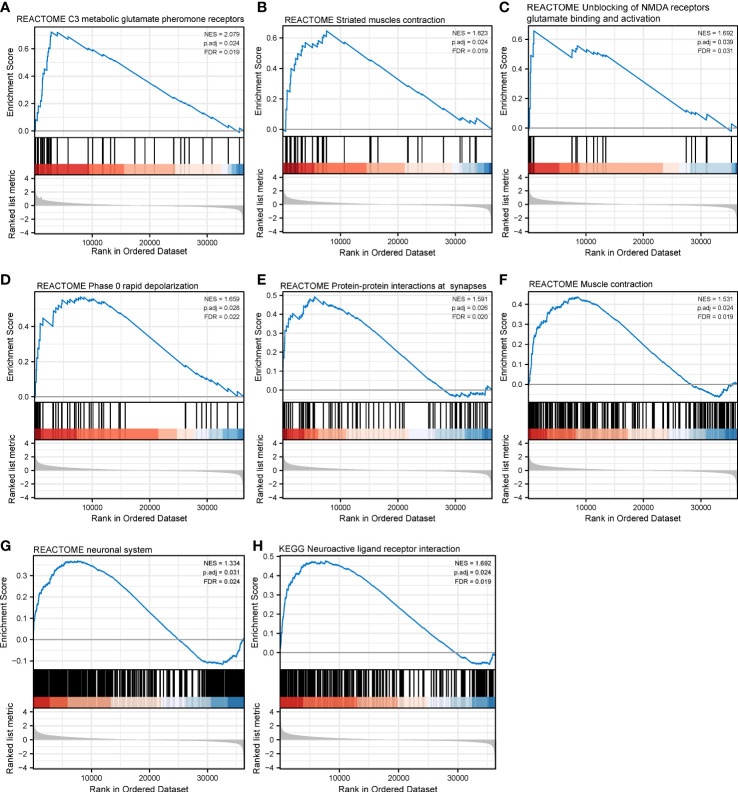
Gene set enrichment analysis of CHRNAG in SKCM. The expression of CHRNG positively correlated with class C3 metabotropic glutamate pheromone receptors **(A)**, striated muscle contraction **(B)**, unblocking of NMDA receptor glutamate binding and activation **(C)**, phase 0 rapid depolarization **(D)**, protein–protein interactions at synapses **(E)**, muscle contraction **(F)**, and neuronal system **(G)** in Reactome gene sets. The enriched KEGG pathways mainly involved neuroactive ligand–receptor interaction **(H)**.

With respect to CHRNG, the significantly enriched Reactome pathways included class C3 metabotropic glutamate pheromone receptors (**Figure 10A**), striated muscle contraction (**Figure 10B**), unblocking of NMDA receptors glutamate binding and activation (**Figure 10C**), phase 0 rapid depolarization (**Figure 10D**), PPIs at synapses (**Figure 10E**), muscle contraction (**Figure 10F**), and the neuronal system (**Figure 10G**). The enriched KEGG pathways mainly involved neuroactive ligand–receptor interaction (**Figure 10H**).

## 4 Discussion

The communications between cancer cells and the nerves are thought to be reciprocal and complex, on the grounds that neuropeptides or neurotrophic factors released by tumor cells promote axonogenesis and neurogenesis to innervate the growing tumor, which in turn, promotes tumor growth, invasion, and metastasis. Additionally, the well-established cancer–immune crosstalk could be extended to cancer–nerve–immune crosstalk. In the case of melanoma, the roles of various neurotransmitters, such as catecholamines, glutamate, serotonin, or cannabinoids, have also been studied. α-MSH and other neurohormones, as well as neuropeptides including substance P, calcitonin gene-related peptide (CGRP), enkephalin, and β-endorphin, have all been implicated as potential factors in the development, growth, invasion, and dissemination of melanoma in a variety of *in vitro* and *in vivo* studies (31).

Based on previous research, our study defined 66 cancer–nerve crosstalk-associated genes through literature review, and unveiled the expression level, mutation features, and functional enrichments of these cancer–nerve crosstalk-associated genes and their association with patient prognosis, immune cell infiltration, and clinical stages in SKCM based on the TCGA database. In queried genes, most mutations happened on GRIN2A, which encoded a member of the glutamate-gated ion channel protein family, an NMDA receptor subunit, underlying the late component of postsynaptic potentials at excitatory synapses. The mutations of GRIN2A in human could induce nervous system diseases such as single-gene epilepsies and schizophrenia (32, 33). However, the effect of GRIN2A mutations in SKCM remains to be elucidated.

KEGG analysis unveiled that key cancer–nerve crosstalk-associated genes were also enriched in the calcium signaling pathway, Ras signaling pathway, and PI3K−Akt signaling pathway, which were implicated to exert an influence in the development and progression of melanoma. Among them, genetic alterations leading to abnormal activations of the Ras signaling pathway represent the most common oncogenic driver mutations, particularly BRAF and NRAS mutations, which occur in approximately 50% and 15% of melanomas, respectively (34). Calcium-related pathways were also involved in the tumorigenesis and progression of melanoma, by influencing not only tumor cells but also the melanoma microenvironment, including immune cells, extracellular matrix, the vascular network, and chemical and physical surroundings (35). PI3K-Akt pathways likewise modulated the biology of melanoma, and targeted inhibitions of the above pathways proved effective in preclinical or clinical settings (36, 37). Hence, the linkages between the cancer–nerve crosstalk-associated genes and these pathways provide further lines of evidence of the potential roles of neural regulation in melanoma.

Then, we constructed a prognostic gene model based on the screened eight genes (GRIN3A, CCR2, CHRNA4, CSF1, NTN1, ADRB1, CHRNB4, and CHRNG). The AUCs of the 1-, 3-, and 5-year ROC curve of this model were 0.704, 0.682, and 0.685 respectively, which were relatively poor. Hence, we took common clinical characteristics in SKCM into account to further build a nomogram, successfully enhancing the prognostic value of the model as the AUCs of the 1-, 3-, and 5-year ROC of the nomogram were 0.850, 0.811, and 0.792, respectively. To our best knowledge, this was the first prognostic model utilizing cancer–nerve crosstalk-associated genes in SKCM.

The nerve system could regulate tumor progression in a diverse array of cellular and molecular processes, including DNA repair, oncogene activation, inflammation and immune response, hematopoiesis, angiogenesis, and apoptosis (20). Among them, manipulating the immune cell activities is of vital importance. Several *in vivo* studies have shown that sympathetic nervous system stimulation of inflammatory signaling can enhance tumor progression and metastasis (34, 35). In the prognostic genes we screened, ADRB1, GRIN3A, CCR2, and CSF1 were universally positively correlated with different immune cells. Stress was indicated as a driver of cancer progression including melanoma. Preclinical and clinical lines of evidence have demonstrated that melanoma showed a positive response to the β1- and β2-adrenoceptor blocker propranolol by inhibiting angiogenesis and disrupting migration of melanoma cells (38–40). The correlation of ADRB1 expression and immune cell signatures implied that the anti-cancer effects of propranolol might be partly through its interaction with tumor-infiltrated immune cells.

These results concerning CCR2 and CSF1 are readily comprehensible considering that CCL2–CCR2 signaling and CSF1-CSF1R signaling markedly participate in cancer immunity. CCL2 released by tumor cells, endothelial cells, fibroblasts, and Schwann cells through engaging with its receptor CCR2 is able to regulate the infiltration and migration of tumor-associated macrophages, which facilitate tumor growth by inducing immune suppression. Overwhelming evidence supported targeting the CCL2–CCR2 axis in various cancer types including in melanoma as candidates for immunotherapy, especially in combination with ICBs, which also echoes our results of correlations between the expression of CCR2 and IC genes (41, 42). Concurrently, interruption of CCL2–CCR2 signaling substantially impaired macrophage-promoted perineural invasion (PNI), which is an ominous event strongly linked to poor clinical outcome (43). This added another rationale for blocking CCL2–CCR2 pathways in view of its complicated roles in tumor, nerve, and immune cells. In addition, β−adrenergic signaling through β-adrenergic receptors could markedly enhance macrophage recruitment into the tumor parenchyma by stimulating tumor cells’ macrophage colony-stimulating factor 1 (M-CSF, encoded by CSF1) production, and further stimulate macrophage expression of transforming growth factor−β (TGF-β), vascular endothelial growth factor (VEGF), IL-6, matrix metalloproteinase 9 (MMP9), and prostaglandin-endoperoxide synthase 2 (PTGS2). Pharmacologic inhibitions of CSF1 signaling could improve the antitumor efficacy of adoptive cell transfer immunotherapy mainly by modulating the macrophage functions (44–46). The results implicated the importance of the interaction of cancer–nerve crosstalk-associated genes with macrophages, which were, to some extent, neglected in the cancer immunotherapies. Furthermore, the extent of correlation of CSF1 with immune checkpoint genes was in concert with recent data implicating CSF1 as a CD8^+^ T-cell-dependent adaptive resistance mechanism to PD-1 blockade and showing that simultaneous CSF1R targeting may be beneficial in melanomas refractory to ICB (47).

In our study, the expression of NTN1 was significantly positively correlated with mast cells, which modulated the cutaneous inflammatory reactions and acted as potential players in different types of skin cancers including SKCM (48, 49). Moreover, the infiltration of mast cells was in direct proportion to the aggressiveness of the tumor. Tumor-infiltrating mast cells were also associated with resistance to anti-PD-1 therapy in a mouse melanoma model (50). The mast cells could release several angiogenic factors and neurotransmitters involved in the evolution of melanoma (51). Netrin-1 encoded by NTN1 promotes the melanoma development coupled with its receptor DCC, which, in contrast, functioned as a tumor suppressor in intestinal cancer and lung metastasis by triggering cancer cell death (52). Moreover, the broad and strong correlation of the GSVA score of the prognostic gene set with immune infiltration also corroborated the delicate interrelationship among cancer cells, nerves, and immune cells.

Finally, we focused on the independent poor prognostic genes CHRNA4 and CHRNG, which respectively encoded a subunit of nicotinic acetylcholine receptor (nAChR). Unlike the relatively consistent cancer-promoting effects of the sympathetic signaling pathway, the parasympathetic nerves showed contradictions in different tumor settings. In a study of breast cancer, activation of the parasympathetic nervous system reduced tumor growth and distant metastasis by decreasing the expression of PD-1 and PD-L1 (53). Parasympathetic neurotomy promoted pancreatic tumorigenesis (54). In contrast, the direct effects of parasympathetic neurotomy in gastric cancer and colorectal cancer patients were reduction of tumorigenesis and decrease of tumor proliferation (10, 55). These contradictory consequences could be explained by inducing sympathetic signaling effects indirectly (56). However, the mechanisms behind the above studies were mostly muscarine acetylcholine receptor (mAChR)-based signaling. Meanwhile, the nAChRs were particularly relevant to lung cancer and nicotine dependence (57, 58). In our study, we identified CHRNA4 and CHRNG as independent poor prognostic genes and performed GSEA on them. The results indicated SKCM patients with different levels of CHRNA4 expression and found that those differential genes based on the CHRNA4 expression level were enriched in retinol metabolism, drug metabolism, plasma lipoprotein remodeling, and the PPAR signaling pathway, which mainly participated in lipid metabolism. In short, CHRNA4 might take part in the metabolic pathway. On the other hand, compared with the low expression of CHRNG, pathways activated in SKCM patients with a high expression of CHRNG mainly concerned muscle contraction activities, which still require further investigation of its relationships with melanoma.

Our work is helpful for future studies in the field of melanoma–nerve–immune crosstalk, as far as we know, this is the first relatively comprehensive analysis of cancer–nerve crosstalk-associated genes that indicates their relationships with immunological features in SKCM. The existence of limitations of our study cannot be ignored. First, the current study was performed primarily based on bioinformatic analyses, which could be further verified by experimental research. Second, the skin tissues of the healthy subjects used as controls are largely different from cancer tissues of SKCM patients, which could bias the results. For example, in experimental situations, both β1- and β2-adrenoceptors were found to be significantly higher expressed in malignant melanoma than benign melanocytic naevi and atypical naevi, which contradicted our analysis (47). Still, our comprehensive bioinformatics analysis of cancer–nerve crosstalk-associated genes in SKCM constructed a valuable prognostic model based on eight genes (GRIN3A, CCR2, CHRNA4, CSF1, NTN1, ADRB1, CHRNB4, and CHRNG) and common clinical characteristics. Our findings could aid further investigation in the molecular mechanisms of the nerve system in the development of SKCM and could help in the search for new therapeutic targets.

## Data availability statement

The original contributions presented in the study are included in the article/**Supplementary material**. Further inquiries can be directed to the corresponding authors.

## Author contributions

FZ and FC contributed to conception and design of the study. FW organized the database. FW performed the statistical analysis. FW and FZ wrote the draft of the manuscript. FC and FZ reviewed and edited the manuscript. FC and FZ acquired the funding. All authors contributed to the article and approved the submitted version.

## Supplementary material

Supplementary Table 1 Cancer-nerve crosstalk associated genes identified from previous reference.

Supplementary Table 2 Expression of 66 cancer-nerve crosstalk associated genes in SKCM and normal skin tissues.

Supplementary Table 3 Univariate Cox analysis of nerve-cancer crosstalk associated genes.

Supplementary Table 4 Risk score of each patient with survival data in TCGA-SKCM cohort based on LASSO regression.

Supplementary Figure 1 Flow chart of our work.

Supplementary Figure 2 The expression heatmap of 66 cancer-nerve crosstalk associated genes in SKCM.

Supplementary Figure 3 The correlation heatmap of all 66 cancer-nerve crosstalk associated genes with 24 types of immune cells.
