# Supplementary figures and images for: Bioinformatic-based genetic characterizations of neural regulation in skin cutaneous melanoma
Source: Front Oncol. 2023 Jun 19;13:1166373. doi: 10.3389/fonc.2023.1166373 (PMC10315675; doi:10.3389/fonc.2023.1166373)

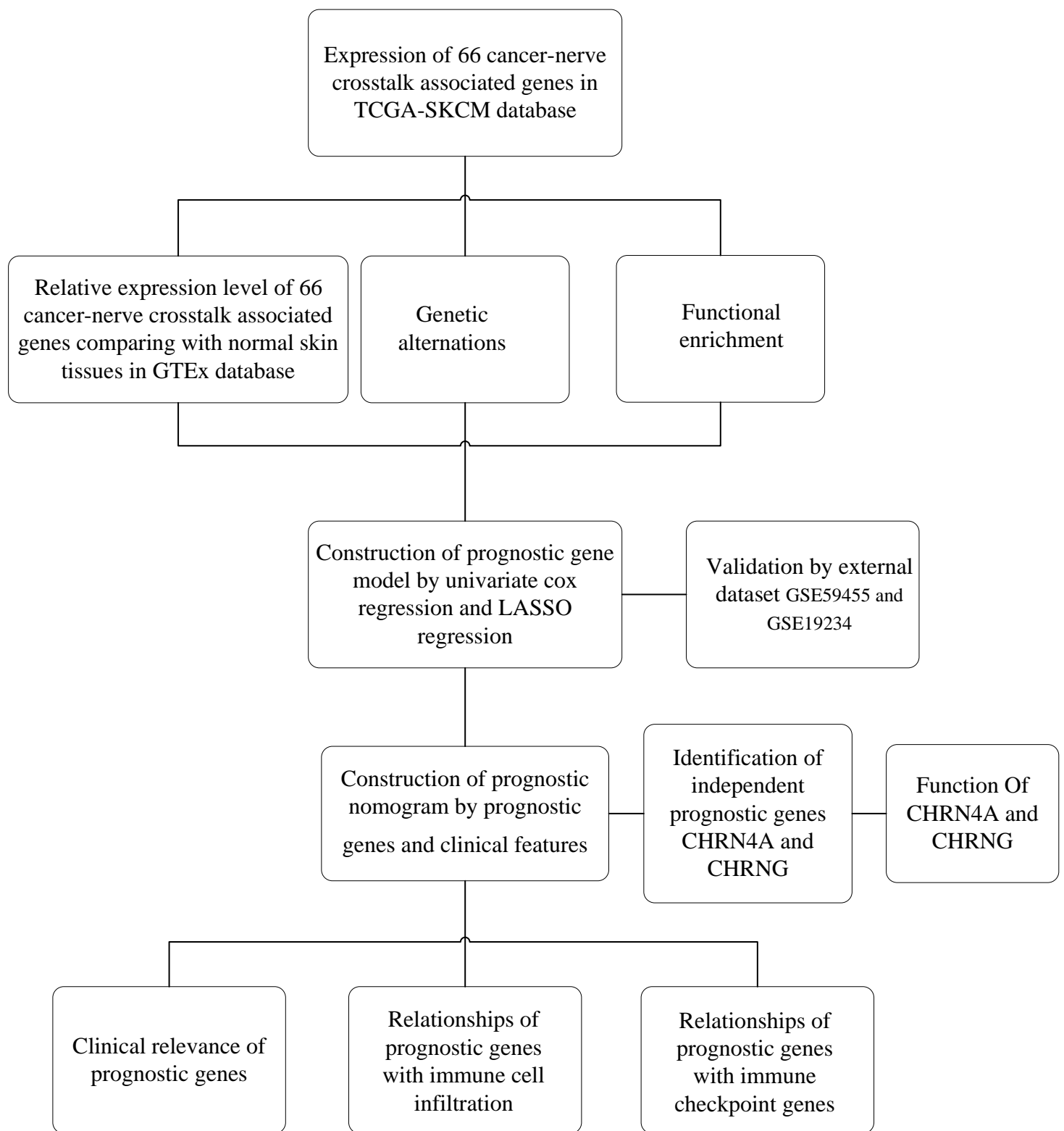

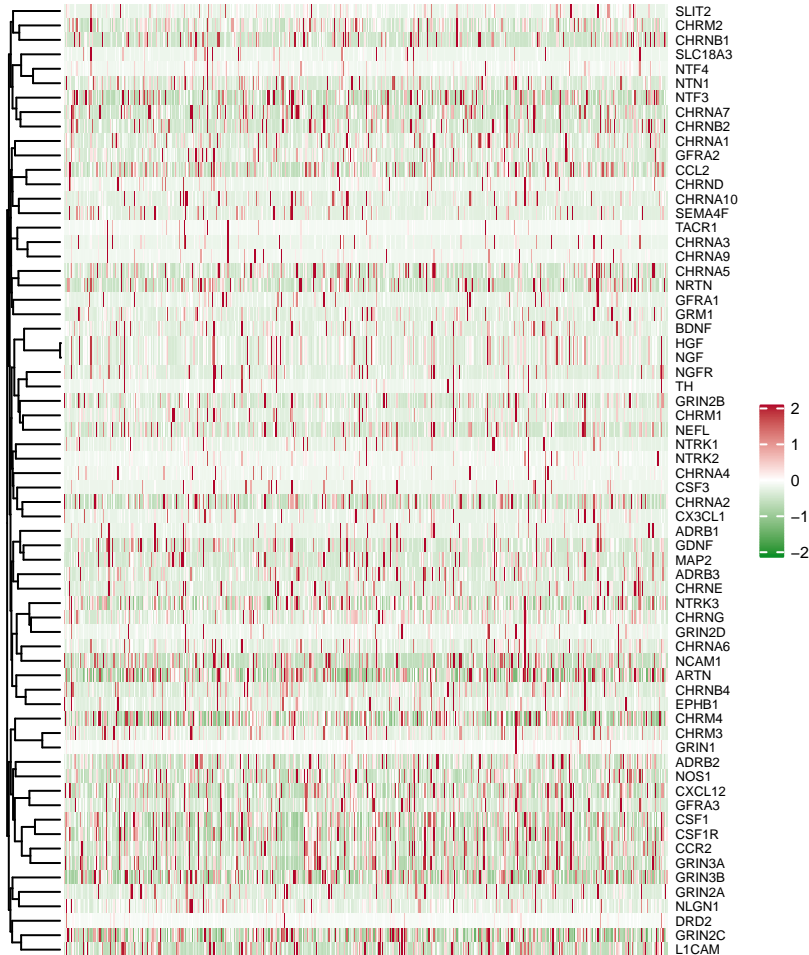

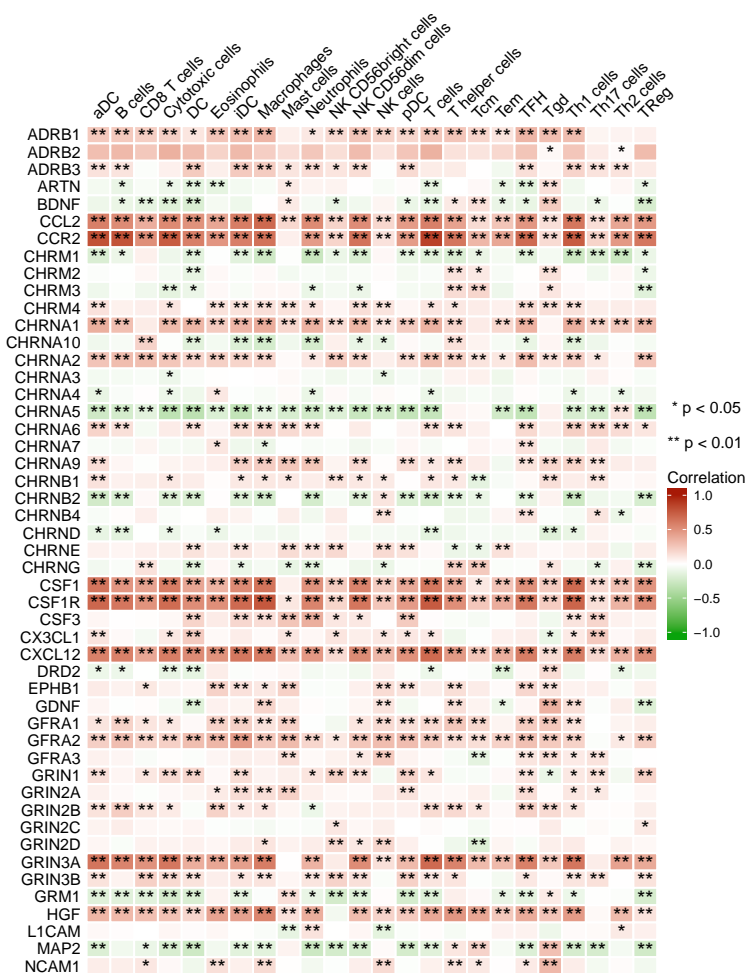

Supplement: Supplementary Figure 1 — Flow chart of our work. [file DataSheet_1.pdf]
